# Sacroiliac Joint and Pelvic Dysfunction Due to Symphysiolysis in Postpartum Women

**DOI:** 10.7759/cureus.18619

**Published:** 2021-10-09

**Authors:** Brian Fiani, Manraj Sekhon, Thao Doan, Brianne Bowers, Claudia Covarrubias, Michaela Barthelmass, Frank De Stefano, Athanasios Kondilis

**Affiliations:** 1 Neurosurgery, Desert Regional Medical Center, Palm Springs, USA; 2 William Beaumont School of Medicine, Oakland University, Rochester, USA; 3 School of Medicine, University of Texas Medical Branch, Galveston, USA; 4 Kentucky College of Osteopathic Medicine, University of Pikeville, Pikeville, USA; 5 School of Medicine, Universidad Anáhuac Querétaro, Santiago de Querétaro, MEX; 6 School of Medicine, California University of Science and Medicine, Colton, USA; 7 College of Osteopathic Medicine, Kansas City University, Kansas City, USA; 8 College of Osteopathic Medicine, Michigan State University, East Lansing, USA

**Keywords:** pregnancy-related complaint, joint disease, etiologies for sacroiliitis, pelvic girdle pain, symphysiolysis

## Abstract

Pregnancy-related pain in the sacroiliac joint (SIJ), lumbosacral region, pubic symphysis, or in any combination of these joints has been coined as pelvic girdle pain (PGP) and has been estimated to affect almost half of all pregnant women. SIJ dysfunction in pregnancy is due to multiple biomechanical mechanisms, such as increased weight, change in posture, increased abdominal and intrauterine pressure, and laxity of the spine and pelvic structures. Moreover, when compared to men, women have increased SIJ mobility due to increased pubic angle and decreased SIJ curvature. These differences may assist in parturition where hormones, such as relaxin and estrogen, cause symphysiolysis. A retrospective review of the literature was conducted in the PubMed database using the search term “pregnancy-related sacroiliac joint pain.” All peer-reviewed studies were included. Around 8%-10% of women with PGP continue to have pain for one to two years postpartum. Patients that were treated with SIJ fusion show statistically significant improvement in pain scores when compared to patients that had non-operative treatment. Although we have a number of studies following patients after sacroiliac (SI) joint fusion for pelvic pain with SI joint dysfunction, further research is needed to study sacroiliac fusion for SI joint dysfunction in postpartum women to better tailor and optimize surgical outcomes for this patient population.

## Introduction and background

The largest true synovial joint in the body, the sacroiliac joint (SIJ), is one of the most common sources of chronic lower back pain (LBP), as it is a highly specialized joint that is innervated by spinal nerves and grants stability, limited flexibility, and support of the upper body [1-3]. Sacroiliitis is defined as the inflammation of the SIJ that can present in both rheumatic and non-rheumatic disorders and can be caused by both traumatic and atraumatic etiologies [2,4]. Consequently, the SIJ has been estimated to account for 15%-30% of chronic low back pain in the general population. Risk factors include obesity, low-grade trauma such as jogging, pregnancy, lumbar fusion, scoliosis, leg length discrepancy, and gait abnormalities [2,3]. The pain can be attributed to the posterior extra-articular elements, such as ligamentous or muscular injuries, enthesopathy, or intra-articular elements [2,3,5].

During gestation and in preparation for birth, the SIJ fibrous apparatus loosens with the presence of hormonal and biomechanical factors, relaxin, estrogen, and symphysiolysis, providing an increase in joint mobility [1,6,7]. Pregnancy-related pain in the SIJ, lumbosacral region, pubic symphysis, or in any combination of these joints has been coined as pelvic girdle pain (PGP) and has been estimated to affect almost half of all pregnant women [6]. While general pelvic gender differences become recognizable as early as the fourth month in utero, SIJ gender dimorphisms do not emerge until the pubescent phases of life and can include certain characteristics that only females possess when compared to their male counterparts. These gender dimorphisms include a narrower sacral angulation, a shorter posterior sagittal diameter of the pelvic outlet, and a narrower sacrum, as well as an iliac bone groove that usually develops in the second decade of life [1]. While pregnancy alone is considered to be an extra-articular pathological cause of SIJ pain, the asymmetry of laxity has been correlated to the presence of pain and clinical symptoms [1,2]. Another cause of pregnancy-related SIJ pain, although rare, is septic sacroiliitis, which is approximated to be 15% of all septic sacroiliitis cases [6]. In the majority of women, the pain resolves within four months after giving birth but persists in approximately 20% of women; PGP that begins during pregnancy without resolving or that develops immediately after pregnancy is termed as postpartum pelvic girdle pain (PPGP) [7].

Although there are no specific historic features, provocation tests, or radiological findings that conclude a definite diagnosis of SIJ pain, the diagnostic algorithm is conducted through a proper medical history followed by physical examination, imaging study, and sometimes diagnostic SIJ blocks [2,3]. There is a wide array of non-surgical treatments including pain medication, steroid injections, physical therapy, individualized pelvic stabilizing exercises, and radiofrequency ablation of the sacral nerves [3,7]. Sacroiliac joint fusion, open or minimally invasive, is reserved for those patients with a diagnosis of chronic SIJ pain who experience pain for a minimum of six months and do not respond to conservative treatment [3]. Currently, there is a scarcity of published outcomes investigating the occurrence of sacroiliac (SI) joint dysfunction in postpartum women. The primary objective of this review was to examine current evidence highlighting the feasibility of SI joint fusion as a treatment modality for SIJ pain in postpartum women. Herein, we examine the literature to date that discusses the risk and correlation of sacroiliitis in postpartum women and current management modalities.

## Review

Pathophysiology of SI joint dysfunction during pregnancy and postpartum

The SIJ assists in transferring weight between the lower body and lumbar spine. There are several ligaments that assist in supporting SIJ movement including the anterior and posterior, sacrospinous, iliolumbar, sacrotuberous, and interosseous sacroiliac ligaments. Muscles that surround the joint include the hamstrings, pelvic floor muscles, erector spinae, psoas, quadratus lumborum, abdominal obliques, and piriformis. They do not act directly on the joint but cross over to produce movement via attachments at the spine or hip. Therefore, SIJ movements are not produced directly but rather through gravity or indirect movements from surrounding muscles acting on the lower extremities or trunk [8].

The female and male sacroiliac joints have numerous biomechanical differences. The female sacrum is wider, more posteriorly tilted, and less curved with a wider sciatic notch and acetabula. These differences may contribute to increased rates of SIJ misalignment in young women [1]. The female SIJ has increased loads, stresses, pelvic ligament strains, and mobility than the male SIJ joint, which may play a role in the higher incidence of pelvic stress fractures and SIJ pain [9]. Compared to men, women have increased SIJ mobility due to increased pubic angle and decreased SIJ curvature. These differences may assist in parturition where hormones, such as relaxin and estrogen, cause symphysiolysis. While these changes may be necessary to give birth, they also cause an increased risk for pelvic pain [1].

SIJ dysfunction in pregnancy is due to multiple biomechanical mechanisms, such as increased weight, change in posture, increased abdominal and intrauterine pressure, and laxity of the spine and pelvic structures. Asymmetric SIJ laxity in pregnancy resulted in three times more risk for developing moderate to severe pelvic girdle pain compared to women with symmetric SIJ laxity. Women with asymmetric SIJ laxity may have a higher risk for chronic pain postpartum [10]. Additionally, changes in weight and exaggerated lordosis result in an anteriorly displaced center of gravity. This causes an increase in SIJ load compounded on the overall laxity of other joints and ligaments, which can further promote the risk of pain and injury [11,12]. Axial spine loading can also compress the intervertebral discs resulting in protrusion and associated back pain [13]. To summarize, pelvic instability, ligament relaxation, asymmetry of the SIJ, and weakness in the pelvic floor, abdominal, and hip extensor muscles are all known contributing factors to continued lower back pain in postpartum women [14].

Pelvic girdle pain and the role of the SI joint

PGP refers to pain in the pubic symphysis, one or both sacroiliac joints, and the gluteal region and is a form of musculoskeletal dysfunction that can affect mothers during both the intra- and postpartum periods, with the point prevalence around 20% [15]. Other studies have shown the prevalence of PGP to range from 4% to 76%, as most definitions of PGP also include pregnancy-related lumbar back pain (PLBP) [12]. The pain is usually between the posterior iliac crests and the gluteal folds, or within the vicinity of the SI joints [15]. This differs from PLBP in that the pain in PGP is not only limited to the lumbar spine, but the pain is also reproducible by clinical tests [15]. Women have described the pain as stabbing, shooting, dull, and burning. The pain in PGP can also be associated with radiation all the way into the calf. Furthermore, PGP is often debilitating and is the most common reason for pregnant women to miss work [16]. A study in Norway performed by Engeset et al. found that these women often feel isolated, lonely, and discouraged [17]. More research is needed to help delineate between PLBP and PGP, as PGP can cause functional disability and have lasting effects, with 8%-10% of women having pain that persists up to two years following delivery [12,15].

There are four main types of PGP: anterior, involving the pubic symphysis; posterior, involving one or both SI joints; miscellaneous, involving anterior and unilateral posterior; and complete, involving all three pelvic joints. A study performed by Robinson et al. showed that out of 46% of women with PGP, 19% reported pain in the anterior pelvis, 14% reported unilateral or bilateral posterior pelvic pain, 4% in the anterior combined with unilateral posterior pain, and 5% had pain in all pelvic joints [18]. The etiology of PGP was first thought to have been due to the activity of the pregnancy hormone relaxin alone, as relaxin’s role is to increase the laxity of ligamentous structures, but that was not found to be significant. There are now proposed mechanisms that use a multifactorial approach, which include trauma, mechanical, hormonal, and degenerative changes [19].

Risk factors for PGP include a history of PGP or low back pain, as well as previous trauma to the pelvis. Factors that have not been found to increase the risk of PGP include age, height, weight, smoking, and contraceptive pills [12,15]. Known risk factors for persistent PGP include increased disability during pregnancy, hypermobility, more than one positive provocation test, and asymmetrical laxity of the SI joints [20,21].

Mechanical considerations of the pelvic girdle

The pelvic girdle’s function is to distribute both the load and force between the upper and lower body, including the spine. This symmetric distribution of force by the pelvic girdle depends on many factors including supporting structures, which are essential for the stability of the SI joints [15]. Stability is also optimized by both form and force closure, which is necessary for the SI joints to transfer the force load to the legs [22]. In pregnancy, a gravid uterus weakens abdominal muscles, thereby increasing the strain on the lumbar muscles. There is also an anterior tilt of the pelvis, shifting a greater load through the SI ligaments [12]. These mechanical changes impair force closure, which in turn decreases the ability of the SI joint to transfer the force to the legs, and increases the shear forces across the SI joints [20,22].

The instability, shear forces, and extra mobility impact ambulation greatly in affected women [15]. The increased laxity of ligamentous structures in pregnancy causes the pelvic girdle to expand and the SI joints to loosen, as well as an increased amount of synovial fluid in the SI joint [15]. However, two studies performed by Damen et al. have confirmed that the amount of SI joint laxity in a woman with severe PGP is not significantly different than in patients with mild pain. These studies show that severe pelvic pain is not related to the amount of laxity, but to the asymmetric nature of the laxity [10,23]. Another study performed by Vleeming et al. showed that 76% of patients with PGP also had pain on palpation of the long dorsal sacroiliac ligament [24]. These findings show that the pain in PGP is heavily influenced by the SI joint and its supporting structures.

PGP clinical provocation tests

PGP is a mostly clinical diagnosis that relies on good history-taking skills that include various orthopedic tests. A European study performed by Vleeming et al. developed guidelines for PGP, which stated that the pain must be reproducible with orthopedic tests [15]. These tests include the posterior pelvic provocation test (P4), Patrick’s Faber test, palpation of the long dorsal sacral ligament, active straight leg raise, and modified Trendelenburg [12,15]. These tests, however, were found to have very low intertester reliability in a study performed by Van Kessel-Cobelens et al. [25]. The examiners agreed on 68% of the findings, but that finding was not significant [25]. CT scans are not recommended to diagnose PGP, although MRI can be used to discriminate between ankylosing spondylitis and PGP. Whereas various orthopedic tests that orient to PGP clinical diagnosis have been proposed, more research is needed to standardize the testing for PGP to make a reliable diagnosis.

The treatment of PGP is limited, as some clinicians do not understand its prevalence and severity. However, some of the treatments that are recommended, according to the literature, include exercises, physical therapy, and pain management. Studies have shown that the use of other treatments including massage, the use of pelvic belts alone, and acupuncture are not useful in PGP [12,15,20]. Therefore, more research is also warranted to better define conservative treatment modalities.

Prevalence of SI joint dysfunction in postpartum

SIJ dysfunction in postpartum women often goes unreported due to the daily demands of motherhood, and they likely only seek medical care when the discomfort significantly impacts daily life. Therefore, SI dysfunction in postpartum women perhaps is more prevalent than previously described. Thus, understanding the potential of continued SI dysfunction during the postpartum period can help healthcare providers educate patients on the signs and symptoms, which could lead to higher rates of interventions.

Pregnancy-related back pain is considered a normal physiologic symptom of pregnancy due to its exceedingly high prevalence [26]. It is predicted that 89% of pregnant women with back pain experience SIJ pain [27]. This distinction may be due to the structural modification of the pelvis that occurs during pregnancy and the postpartum period. The modifications are induced by the peptide hormone, relaxin, which is responsible for collagen remodeling that allows for joint laxity and ultimately the expansion of the pelvis [1,23,26-29]. The laxity of the joints often occurs in an asymmetric pattern; thus, the imbalance of forces placed on these joints may lead to SIJ dysfunction that continues into the postpartum period [1,23,28].

Damen et al. state that pregnant women with asymmetrical laxity of the pelvis have a three-fold increased risk of postpartum SIJ dysfunction when compared to pregnant women with symmetrical laxity. Of note, lordosis, weight gain, and trauma of childbirth, such as bleeding into the joint during delivery, may increase the risk of sacroiliitis [29]. Sipko et al. examined the function of the SIJ among women in their eighth month of pregnancy and three months after childbirth. The use of the Patrick test showed 36% of women had pain in the SIJ region during pregnancy and 23% of those women had continual pain postpartum [26]. Additionally, the standing flexion and standing Gillet test showed that 70% of women had SIJ disorders during pregnancy and the postpartum period. Ghodke et al. conducted a cross-sectional study that showed 26% of postpartum women suffered from SIJ dysfunction [27]. The highest prevalence was seen in full-term normal delivery compared to lower segment cesarian sections [27].

Prognostic factors of SI joint dysfunction

PGP differs from LBP in that the latter occurs as a dull pain in the lumbar region, while PGP is usually present between the posterior iliac crest and gluteal fold, with potential radiation down the thigh [30,31]. Furthermore, it is estimated that around 8%-10% of women with PGP continue to have pain for one to two years postpartum [30,32,33]. In a systematic review, Wuytack et al. examined 26 potential prognostic factors for PPGP across three prospective studies, two of which studied pelvic girdle syndrome (PGS), which is defined as pain in both SI joints and the symphysis pubis. They found women with pain in three to four areas of the pelvic girdle had stronger pain intensity 12 weeks postpartum and were less likely to recover. Women with a body mass index (BMI) of 25 or more and women with a history of pre-pregnancy low back pain had a higher disability and were more likely to have persistent PPGP 12 weeks after childbirth. Women who used crutches in pregnancy, experienced pain in three pelvic girdle locations compared to one or two, underwent an instrumental birth instead of an unassisted vaginal birth, received cesarean section, had a co-morbidity index of two to three or four or more, had a BMI of 30 or more, presented with a history of low back pain, occasional smoking, menarche onset of 10 or younger, and experienced emotional distress at one point during pregnancy were more likely to have persistent and severe PGS at six months postpartum [34]. Additionally, factors such as prolonged labor, a high level of pain provocation tests, a lower mobility index score, and early gestational pain are correlated with long-term PGP.

SI joint fusion and clinical outcomes

The majority of patients with PPGP have symptoms that are self-limiting; however, if they persist, the aforementioned treatment options are viable [15]. If conservative therapies fail, minimally invasive SI joint fusion is an available treatment modality for postpartum women with SI joint dysfunction. There is a paucity of studies examining SI joint fusion in women with PPGP; however, Capobianco et al. in the Sacroiliac Joint Fusion Investigation (SIFI) Study Group studied a sub-group with SI joint disruption and/or degenerative sacroiliitis in a prospective, multicenter trial of SI joint fusion. Patients with PPGP were significantly younger than women without PPGP and men (43.3 vs. 52.5 and 50.7 years, respectively). Interestingly, there was no difference in BMI, duration of pain, and previous lumbar spinal fusion between PPGP and non-PPGP women. Overall, PPGP women had improved pain, function, and quality of life scores after SI joint surgery. With the mean visual analog scale (VAS) pain level being 81.9, pain in this PPGP group lowered to 51.1. Oswestry Disability Index (ODI) decreased from 52.2 at baseline to 30.4 at six months and 32.8 at 12 months. Physical component score (PCS) increased from 32 at baseline to 41.6 at 12 months. Mean EuroQol 5 Dimensions (EQ-5D) time trade-off (TTO) utility score improved from 0.42 to 0.72 at 12 months. Almost all patients were somewhat or very satisfied with surgery at one-year follow-up. Mean total adverse events were comparable between groups. The revision rate was higher at 5% in PPGP women vs. 2% in non-PPGP women [7]. While this study is promising, clinical outcomes of SI joint fusion have been better studied in patients with pelvic pain.

Furthermore, Nystrom et al. followed 49 patients after anterior SI joint fusion and found 26 patients had significantly lower levels of pelvic pain than before, 28 reported a higher quality of life, and 26 reported better sleep post-operatively [35]. In another study, Buchowski et al. found in 20 patients who failed nonoperative treatment multiple etiologies of sacroiliac symptoms, 13 of which had SI joint dysfunction. A total of 17 patients (85%) achieved solid fusion, and significant improvements were seen from the SF-36 Short-Form including physical functioning, bodily pain, vitality, social functioning, emotional roles, and neurogenic and pain indices [36]. In a randomized controlled trial, Polly et al. assigned 102 patients to minimally invasive SIJ fusion with triangular titanium implants and 46 patients to nonsurgical management. At six months, the SIJ fusion group more often achieved the primary success endpoint, consisting of improvement in VAS SIJ pain alongside no serious device-related adverse events or reoperation by six months, than the nonoperative group (81.4% vs. 26.1%; Bayesian posterior probability of superiority >0.9999). By 12 months, VAS SIJ pain scores defined as improvements from baseline of at least 20 points were 81.6% vs. 12.5% (P < 0.001) for SIJ surgery patients and nonsurgical patients, respectively. ODI score improvement defined as an increase of 15 points from baseline was 72.4% vs. 10.0% with SIJ surgery patients vs. nonsurgical patients (P < 0.05). In addition, SF-36 and EQ-5D TTO values showed significant improvements in the SIJ fusion group over the nonsurgical management patients [37]. We can see here that SI joint fusion can be utilized for patients who fail conservative management with positive outcomes along with a sound clinical judgment. Although we have a number of studies following patients after SI joint fusion for pelvic pain with SI joint dysfunction, further research is needed to study sacroiliac fusion for SI joint dysfunction in postpartum women to better tailor and optimize surgical outcomes for this patient population.

## Conclusions

The SI joint in the pregnant and postpartum female is susceptible to dysfunction due to multiple biomechanical changes that happen over the course of gestation. This includes an increased angle of lordosis, weight gain, and structural trauma due to the physiologic process of childbirth as examples. The aforementioned biomechanical changes, along with hormonal fluctuations through each trimester, particularly with increased levels of estrogen and relaxin, have been found to decrease the stability of the joint leading to sustained postpartum back pain that may necessitate surgical intervention if conservative treatment fails.

The present review contributes to the furthering of our understanding of SIJ joint dysfunction in pregnant and postpartum women. Taken together, the literature suggests that due to the relative frequency of SI joint dysfunction in pregnant and postpartum women, future applications toward understanding and treating the dysfunction should seek to identify the pathology before symptoms manifest based on provocation and diagnostic maneuvers. These findings have implications in the clinical setting by elucidating the advantages of measuring levels of estrogen and relaxin, which could provide both academic and clinical benefits. Moreover, we were able to highlight that physical therapy has proven to be incredibly efficacious, with both rehabilitation therapy and home exercise programs found to alleviate chronic pain via correction of biomechanical instability. Moreover, the use of proper fitting of an SI joint belt, as well manual medicine (osteopathic manual manipulation) have also been found to improve stability and pain reduction. We conclude that there is a need for studies that examine these treatments in the context of SI joint dysfunction in a controlled fashion to provide sufficient data to warrant clinical efficacy. Additional studies with larger cohort sizes and adequate controls will allow for a better understanding of the role of manual medicine treatments in the context of SI joint dysfunction.
